# Dynamics of tear fluid desiccation on a glass surface: a contribution to tear quality assessment

**DOI:** 10.1186/0717-6287-47-25

**Published:** 2014-06-04

**Authors:** Leonidas Traipe-Castro, Daniela Salinas-Toro, Daniela López, Mario Zanolli, Miguel Srur, Felipe Valenzuela, Aníbal Cáceres, Héctor Toledo-Araya, Remigio López-Solís

**Affiliations:** Fundación Oftalmológica Los Andes Ophthalmology Clinic (FOLA), Vitacura, Santiago, Chile; School of Medical Technology-Ophthalmology, Faculty of Medicine, University of Chile, Santiago, Chile; Cellular and Molecular Biology Program, Faculty of Medicine-ICBM, University of Chile, Independencia 1027, Postcode 8380453 Santiago, Chile

**Keywords:** Tear, Tear ferning test, Dry eye, Dark-field microscopy

## Abstract

**Background:**

Fern-like crystalloids form when a microvolume of tear is allowed to dry out at ambient conditions on a glass surface. Presence of crystalloids in tear “microdesiccates” is used to evaluate patients with Dry-Eye disease. This study aims to examine morphologically the desiccation process of normal tear fluid and to identify changes associated with accelerated tear evaporation. Tear microdesiccates from healthy (Non-Dry Eye) and Dry Eye subjects were produced at ambient conditions. Microdesiccate formation was monitored continuously by dark-field video microscopy. Additionally, accelerated desiccation of tear samples from healthy subjects was conducted under controlled experimental conditions. Particular morphological domains of tear microdesiccates and their progressive appearance during desiccation were compared*.*

**Results:**

In normal tear microdesiccates, four distinctive morphological domains (zones I, II, III and transition band) were recognized. Stepwise formation of those domains is now described. Experimentally accelerated desiccation resulted in marked changes in some of those zones, particularly involving either disappearance or size reduction of fern-like crystalloids of zones II and III. Tear microdesiccates from Dry Eye subjects may also display those differences and be the expression of a more synchronous formation of microdesiccate domains.

**Conclusion:**

Morphological characteristics of tear microdesiccates can provide insights into the relative rate of tear evaporation.

## Background

Fern-like microcrystalloids are formed when some biological fluids are placed as sessile microdrops on glass surfaces and allowed to dry out at ambient conditions [1–3]. In the case of tear fluid, failure in the formation of fern-like structures has been closely associated with Dry Eye disease [4–6]. Up-to date, the Tear Ferning test using the Rolando’s scoring system has proven useful as a laboratory aid in Dry Eye assessment [4, 7–9]. Accordingly, abundance of fern-like tear crystalloids is suggestive of normality (scores I and II) whereas reduced tear ferning (scores III and IV) is usually observed among Dry Eye patients [5, 6, 10]. Based on our experience with over six hundreds tear ferning assays, desiccation of a tear sessile drop collected from a healthy subject on a glass surface results in circular tear “microdesiccates” comprising four discrete morphological domains or zones, namely, an outer structured hyaline zone I, a band of regularly and centripetally oriented crystalloids that are distinctive of zone II, a central zone III comprising typical randomly distributed fern-like structures differing to each other in robustness, length and branching, and, finally, a transition band, which is a noticeable structure located between zones I and II that seems to serve to anchor the zone II ferns [11]. All these structural or morphological components of a tear “microdesiccate”, besides the fern-like crystalloid structures, may also be the expression of a normal tear composition and, probably, a normal tear film.

A number of reports have shown that tear protein profiles may exhibit major alterations when the tear fluid subjected to desiccation has been sampled from Dry Eye patients [12, 13]. On the other hand, changes in lipid composition or mucin composition of tear fluid resulting from dysfunctional Meibomium glands or from goblet cell deficiency, respectively, have been associated with Dry Eye [14–18]. Thus, the reduced ability to organize tear ferns can be associated with changes of diverse origin in the tear composition. At least some of those biochemical changes may affect the ability of the tear fluid to retain water as it might occur in patients expressing the evaporative subtype of Dry Eye [19, 20]. Accordingly, it would be expected that the formation of fern-like structures during desiccation of microvolumes of tear fluid on glass surfaces may be also influenced by or be an expression of the rate of tear evaporation. The present study was aimed at characterizing the progression of desiccation of tear fluid from healthy subjects (absence of Dry Eye) on a glass surface using a morphological approach. The study also aimed to evaluate the effect of various experimental conditions that enhance evaporation of tear samples collected from single healthy subjects both on the dynamics of desiccation and the morphological features of the resulting tear microdesiccates. Finally, the study characterized morphologically a differing progression of desiccation of tear fluid collected from both eyes of a mild/moderate Dry Eye case.

## Results

### Rate of tear desiccation

**Figure 1:**
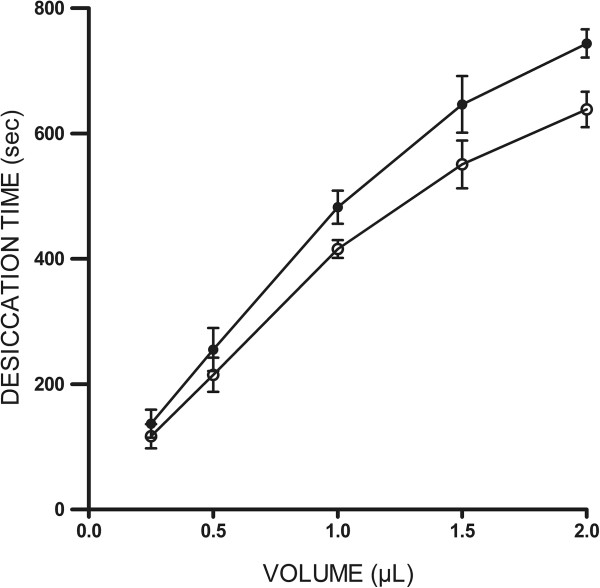
**Time required for complete desiccation of tear microvolumes on a glass surface.** Aliquots of a single tear sample collected from a healthy donor were allowed to dry at ambient conditions Volume of tear and corresponding time for full desiccation were related linearly (r = 0.997). Times for desiccation of equivalent aliquots of water (o—o) and tear (•—•) were statistically similar (p > 0.05, Mann–Whitney). Data are representative of three independent experiments.

Under the current experimental conditions in this study, full desiccation of 1 μL aliquots of tear fluid collected from healthy (non Dry Eye) donors usually took around 9–10 minutes. A linear relationship between volume of tear sample in the range 0.25-2 μL and the time necessary for complete desiccation was observed. In this volume range, desiccation of tear fluid took consistently 15% longer than desiccation of equivalent aliquots of water. Such difference, however, was not statistically significant (p = 0.2738, Mann–Whitney) (Figure 1).

### Tear microdesiccates from healthy subjects display a similar four-domain design

**Figure 2:**
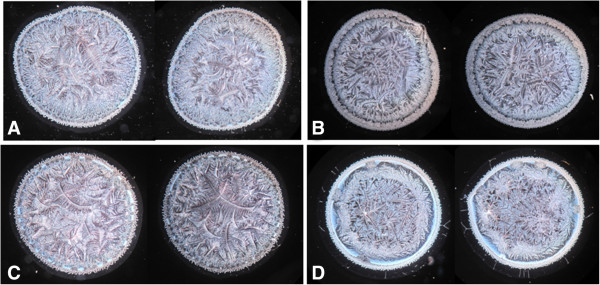
**Intraindividual similarities and interindividual differences in the morphological appearances of tear microdesiccates from single healthy subjects.** Microdesiccates from 1-uL aliquots of two successive samples of tear fluid taken from single healthy subjects (same eye) displayed marked morphological similarities (neighbour images). Microdesiccates corresponding to four subjects **(A, B, C and D)** are shown. Note also some clear differences among microdesiccates from different subjects, although a general design is maintained. Microdesiccates in the figure were rated I-II (normal) in the conventional Rolando’s classification system.

**Figure 3:**
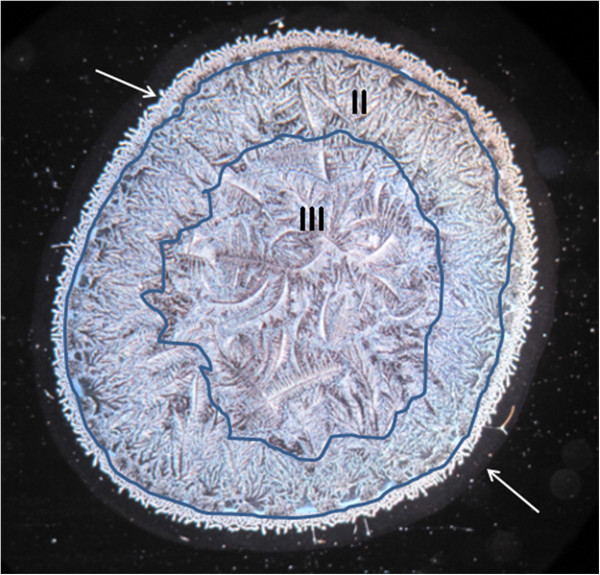
**Representative morphological pattern of a normal tear microdesiccate.** Typical four-domain structure of a tear microdesiccate from a healthy individual. Those domains have been named respectively as zone I (the outermost, lower arrow), transition band (upper arrow), zone II (centripetally-oriented crystalloids) and zone III (major crystalloids at the center). Both zones II and III and their boundaries have been demarcated.

Multiple microdesiccates produced from identical aliquots taken from a single sample of tear fluid were strikingly reproducible in terms of size, design and shape of structural elements. By contrast, tear microdesiccates produced simultaneously from identical aliquots of tear fluid sampled from different healthy subjects usually exhibited some marked differences in discrete structural components (Figure 2). Those differences were sex- and age-independent. However, all the tear microdesiccates shared a common general design comprising a four-domain organization. For convenience those domains have been called zones I, II and III and transition band. Zone I is the outermost band of amorphous material that surrounds the whole circular area of the desiccate. Zone II is a dense band of multiple and identical fern-shaped and leaf-shaped crystalloid structures, which is adjacent to zone I. Zone III, the central zone of the desiccated tear sample, presents typical fern-like crystalloid structures displaying a variety of forms differing to each other in robustness, length and branching. Finally, the transition band is a structurally discrete and usually continuous domain located along the whole interphase between zone I and zone II (Figure 3).

### Temporal appearance of the morphological zones occurring in a normal tear microdesiccate

**Figure 4:**
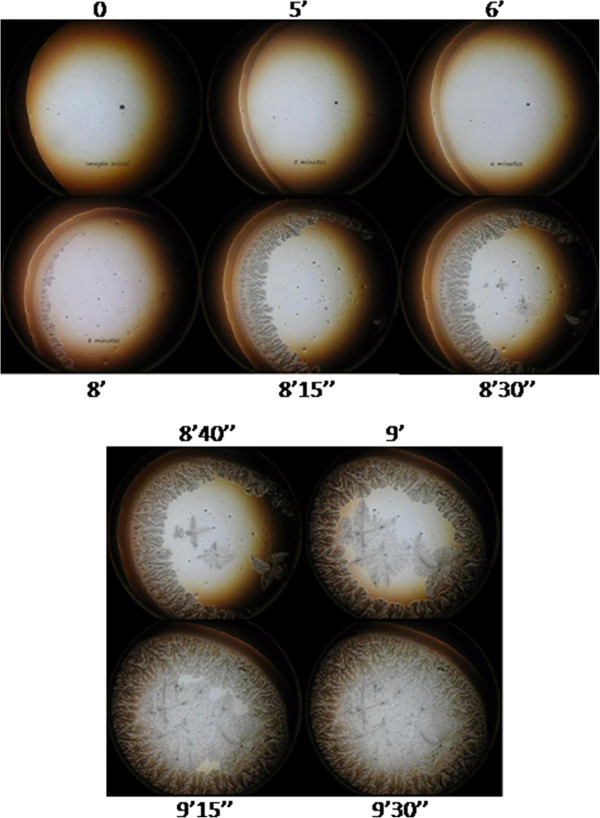
**Progressive appearance of the morphological domains of a tear microdesiccate from a healthy subject.** One-uL aliquots of tear fluid from a healthy subject became fully desiccated in about 9–10 min. Zone I was the first morphological structure to appear progressively during the first half of the time needed for full desiccation. Then, crystalloids of zone II started to appear and grow within a narrow period of time from a number of sites located close to zone I. While crystalloids of zone II were growing centripetally, crystalloids of zone III started to grow multidirectionally from irregularly distributed nucleation sites located close to the center. Physical contact between the growing apices of single crystalloids of either zones II or III with any other crystalloid structure resulted in halting of their growth process (contact inhibition). Desiccation ended when all the zone II and zone III crystalloids became contact inhibited. Times elapsed since the start of desiccation are indicated. One experiment representative of six is shown.

Under the ambient conditions in this study, 1 μL of tear was fully desiccated in about 9–10 minutes (desiccation time). Under the dark-field microscope, zone I was the first domain to become unequivocally identifiable. After appearing simultaneously as a thin strip along the whole perimeter of a roughly circular area of the sessile drop of tear, zone I became progressively thicker and reached its maximum width during the first half of the time needed for full desiccation of the tear aliquot, that is, around 4–5 minutes. Then, following a stage of apparent rest spanning most of the second third of the whole time of desiccation, a series of major and rapid crystallization events started to take place. Formation of the zone II crystalloids was clearly the first major noticeable change at this third stage of tear desiccation. These crystalloids started to form by growing centripetally from regularly spaced sites located on a unstructured but distinct narrow strip in proximity to zone I. While zone II crystalloids were still growing, two events took place at a fourth stage. Firstly, the unstructured narrow strip at the interface between zone I and zone II became more structured and, secondly, a few irregularly disperse sites located close to the center of the tear fluid under desiccation seemed to serve as nucleation points on which morphologically distinct fern-like crystalloid structures began to grow multidirectionally. Each of the branches of these zone III crystalloids grew continuously until they made contact with other growing crystalloid structures from either zone II or zone III. Complete convergence of all the growing crystalloids from zones II and III marked the endpoint of tear desiccation. An usually clear-cut boundary between zones II and III became the most visible morphological evidence of this contact inhibition phenomenon (Figure 4).

### Effect of experimentally accelerated tear desiccation on the four-domain morphology of tear microdesiccates

**Figure 5:**
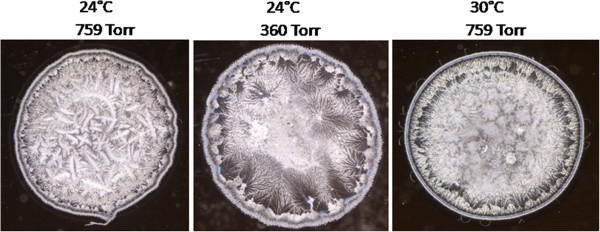
**Changes in the morphological features of tear microdesiccates produced under conditions of accelerated evaporation.** One-microliter aliquots from a single sample of tear collected from a healthy subject were allowed to dry simultaneously either at ambient conditions (left), at reduced pressure (center) or at a higher temperature (right). Under both non-ambient conditions, the time of desiccation was reduced by half and the resulting microdesiccates lacked distinctive zones II and III, preserved the surrounding zone I and exhibited much smaller and more homogeneous groups of crystalloids than those observed in the distinct zones II and III of the control microdesiccate. Figures are representative of five independent experiments.

Rate of water evaporation depends on a number of environmental factors, such as atmospheric pressure, water vapor pressure and temperature. Thus, it would be expected that the rate of water evaporation from a sample of tear placed on a glass surface would increase either by lowering the air pressure surrounding the sample, by removing water vapor from the environment of the sample or by increasing the air temperature. In order to correlate the drying rate of a tear sample with the morphology of the corresponding tear microdesiccate, in a first experiment two 1-uL aliquots drawn from a single tear sample collected from a healthy subject were placed on two independent glass surfaces (microscope slides). One of the aliquots was allowed to dry at ambient temperature (24°C), air pressure (759 Torr) and relative humidity (41%) while the second aliquot was allowed to dry under reduced air pressure (360 mm Hg/Torr) inside of a 45 mL Falcon disposable tube. Both samples were placed at a distance of 10 cm. Under these conditions, the time of desiccation of the tear aliquot placed in the Falcon tube was reduced by half. Morphological analysis of both tear microdesiccates showed marked differences. In contrast with the usual four zones occurring in normal tear microdesiccates produced at ambient conditions, the tear microdesiccate obtained at reduced air pressure showed a significantly thinner zone I, a roughly normal transition band, a paucity of disperse small-sized crystalloids and a central zone strikingly devoid of major crystalloids (Figure 5). In an analog experiment, two 1-uL aliquots drawn from a single tear sample taken from a healthy subject were placed on two independent glass surfaces. One of the aliquots was allowed to dry at the same ambient conditions indicated above (24°C, 759 torr) while the second aliquot was allowed to dry in an oven at 30°C. In this experiment, time needed for full desiccation of the tear sessile drop in the oven was also reduced by half. Again, both tear desiccates showed marked morphological differences. The abundant fern-like crystalloids occurring in zone II and zone III of the tear desiccate produced at ambient temperature, at 30°C were replaced by abundant groups of small crystalloids making contact with each other. In addition, at this higher temperature, differentiation of zones II and zone III disappeared. By contrast, the translucid zone I maintained its main characteristics at both temperatures. Thus, substitution of the typical zone II and zone III crystalloids by roughly uniform small but abundant crystalloids was a common observation in microdesiccates produced under various conditions of accelerated desiccation (Figure 5).

### Temporal progression of the formation of tear microdesiccates from cases of patients with Dry Eye

**Figure 6:**
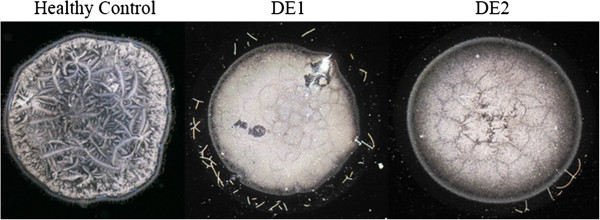
**Tear microdesiccates from Dry Eye patients.** One-μL aliquots of tear fluid collected from two moderate or severe Dry Eye patients were subjected to desiccation on glass slides at ambient conditions. **Patient DE1:** Woman, 40 y.o., contact lens wearer for 25 years, OSDI score 25, Cochet-Bonnet aesthesiometry 45, borderline slit-lamp biomicroscopy (reduced tear meniscus height, meibomitis), tear break-up time test 9 sec, Schirmer test 7 mm. Diagnosis: moderate Dry Eye. Time for tear desiccation: 4 min 50 sec. **Patient DE2:** Man, 72 y.o., diagnosed with rheumatoid arthritis, OSDI score 54, altered slit-lamp biomicroscopy (+++ bilateral punctate staining of bulbar conjunctivas with fluorescein), tear break-up time test 4 sec, Schirmer test 3 mm, Jones test 0 mm, punctal occlusion for 6 years. Diagnosis: severe Dry Eye. Time for tear desiccation: 5 min 15 sec. Note in both tear microdesiccates a common central zone comprising abundant small and homogeneous contact-inhibited crystalloids, which evoke tear microdesiccates produced under conditions of accelerated desiccation. For comparison, a tear microdesiccate from a healthy volunteer is shown (left).

**Figure 7:**
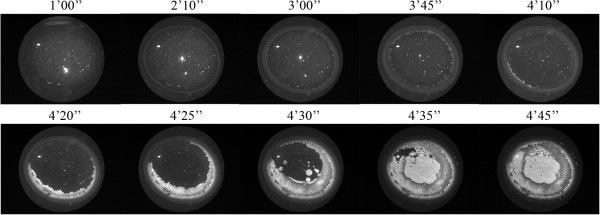
**Dynamics of microdesiccate formation in a tear sample collected from a Dry Eye patient (left eye).** Tear fluid was collected separately from each of both eyes of a mild/moderate Dry Eye patient: Woman, 26 y.o., OSDI score 18.75, tear break-up time test 7 and 8 sec*, Schirmer 15 and 17 mm*, tear ferning score III and III* (*right and left eyes, respectively), bilateral cornea punctata. One-μL aliquots of each tear sample were subjected to desiccation at ambient conditions. At various times during desiccation video-images were captured. Zone I was the first morphological structure to be identified during the first half of the total desiccation time. Then, within a few seconds layers of small and diverse crystalloids were formed centripetally and desiccation ended when many tiny crystalloids grew almost synchronously from many nucleation sites located all over the central area before becoming contact-inhibited. These tiny crystalloids were the core of a honeycomb pattern in the desiccate. Time elapsed since the start of desiccation is shown on top of each figure. This whole series of events was highly reproducible in at least five independent assays per eye. Representative sequence corresponding to the left eye is shown.

**Figure 8:**
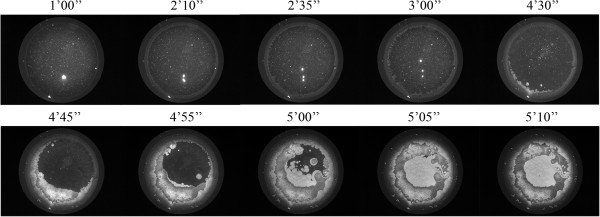
**Dynamics of microdesiccate formation in a tear sample collected from a Dry Eye patient (right eye).** The Dry Eye patient and the experimental assessment of tear desiccation are described in the legend to Figure 7. In this case, the representative sequence of photographs corresponds to desiccation of tear fluid from the right eye.

Dry Eye has been frequently associated with tear fluid displaying a lower tendency to form ferns upon desiccation on glass surfaces [5, 6, 10]. Figure 6 shows desiccates of microvolumes of tear samples taken from two random patients with moderate or severe Dry Eye. Times needed for full desiccation of those tear samples were in average 40% shorter than those observed in desiccation of a tear sample from a healthy subject (p < 0.01). Samples from both patients showed several marked changes in the four-domain structure, the most remarkable and common of which was the disappearance of the distinctive zones II and III together with the formation of abundant groups of small crystalloids making contact with each other. Such remarkable differences evoked the morphological features of tear microdesiccates from healthy subjects produced under experimental conditions facilitating desiccation. Thus, tear samples collected separately from both eyes of a mild/moderate Dry Eye patient were subjected to video-imaging during tear desiccation. As in the production of microdesiccates from normal tear fluid, zone I was the first visible structure to appear during desiccation of both tear samples of the Dry Eye patient. At variance with progression of microdesiccation of tear from healthy subjects, however, in both tear samples from the Dry Eye patient a variety of crystallization events started to take place simultaneously all over the area of desiccation soon after zone I was apparently complete. Layers of tiny and small centripetally oriented crystalloids started to form and progress rapidly supported on the zone I-transition band complex. Within a few seconds, in the most central area under desiccation many groups of small or tiny fern-like or leaf-like crystalloids started to grow multidirectionally. These crystalloids grew until they entered in contact with neighbor groups of crystalloids of the same morphological characteristics. The endpoint of desiccation occurred when all the growing groups of microcrystalloids halted their growth (Figures 7 and 8). Altogether, the resulting snowflake or honeycomb appearance of the central area of these tear microdesiccates seems to be the consequence of the characteristic organization of multiple foci of small crystalloids growing synchronously until contact inhibition takes place.

## Discussion

In this study we have described the dynamics of formation of a domain-based structure produced by desiccation of a sessile tear drop on a glass flat surface. Overall, our observations indicate that each fern-like or leaf-like crystalloid in the tear microdesiccate is formed from an origin site and that its orderly lineal growth and branching end when the growth frontlines of the crystalloid get in contact with other forming or already formed crystalloid.

Spontaneous desiccation of a microvolume of tear fluid collected from healthy subjects gives rise to tear microdesiccates consisting of four domains named zones I, II, III and transition band [11]. We have now shown that those four domains of a normal tear desiccate are formed asynchronously. The outermost hyaline zone I of the tear microdesiccate is the first domain to form at an early stage of desiccation. This structure represents a “pinning” stage of the desiccation process by which the sessile drop of the tear fluid becomes firmly bound to the glass surface thus preventing a decrease in the contact surface between the tear and the glass. Later, during a second stage of the desiccation process, water loss occurs at the water/air interface while the tear drop would be undergoing successive changes of shape [25, 26]. During this period, only some degree of disordered movement can be hardly seen by dark-field microscopy in the fraction of tear that remains in a liquid or semiliquid state. At the onset of the third stage, from specific points usually located at roughly regular distances close to the hyaline zone, fern-like or leaf-like crystalloids of zone II start to grow unidirectionally towards the center of the circular area of desiccation. While zone II crystalloids are still experiencing its unidirectional centripetal growth, disperse crystalloids start to appear asynchronously near the center of the desiccation area (zone III). Each of these nascent zone III crystalloids emerges from a single site, is usually multibranched and becomes larger, less regular in structure and more robust than zone II crystalloids. The highly diverse extents of these zone III crystalloids, and the ones of their branches, seem to depend stochastically on the contact inhibition phenomenon. Formation of zone III crystalloids would represent a fourth and final stage of the tear desiccation process. Contact between zone II and zone III crystalloids inhibits their growth and demarcates the end of the organization of a tear microdesiccate. Overall, formation of zones II and III are late, separate and partly overlapped events during desiccation of normal tear. Their formation in the final stages of desiccation occurs much faster than the visible events of the previous stages of tear desiccation. Anyhow, formation of a tear microdesiccate from any normal tear fluid sample seems to occur in an orderly sequence of events.

Formation of tear crystalloids can reflect the presence of definite components in the tear fluid, mostly proteins, mucins and salts [27, 28]. In support of that view and regardless of significance for desiccation that those tear components may have, in this study we have given some evidence showing that tear microdesiccates from any single subject are essentially identical whereas tear desiccates produced from tear samples provided by different subjects can vary markedly. However, the morphological characteristics of tear desiccates produced from a single tear sample are dependent not only upon the composition of the tear fluid but also on the conditions under which desiccation takes place [29]. In this study we showed that a single sample of normal tear may produce morphologically different microdesiccates under different drying conditions. Thus, under drying conditions that enhance tear water evaporation, such as reduced air pressure, reduced partial water pressure or higher ambient temperature, crystalloids would be originated from a higher number of origins. Because the growth of single crystalloids seems to occur continuously until they make contact with other forming or already formed crystalloids, then a faster desiccation will result in smaller crystalloids. Altogether, these experimental observations suggesting a link between morphological features of tear microdesiccates and relative rate of tear evaporation may provide an additional source of valuable information on tear evaporation from single eyes that may help to assess and diagnose ocular surface disorders [4, 7–9, 12, 18, 23, 30–36]. In our view, the abundance of small crystalloids instead of the vigorous zone III crystalloids in a tear microdesiccate would represent the morphologic expression of a rapid evaporation of the tear water. In this regard, images of honeycomb structures occurring in tear ferning tests of Dry Eye patients, as reported by other laboratories [29], would be the expression of abundant groups of small crystalloids whose growth from a high number of origin sites was rapidly halted by their contact with similar growing neighbor groups of crystalloids.

**Figure 9:**
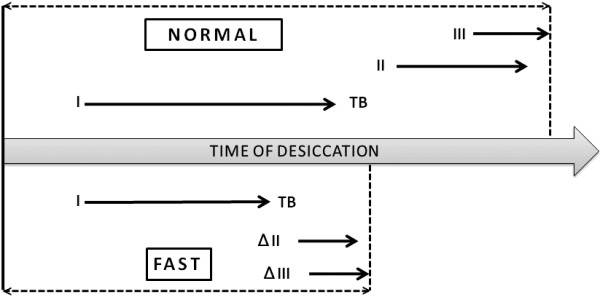
**Diagram representing the stepwise appearance of the morphological domains of a normal tear microdesiccate and its probable modification in patients with evaporative Dry Eye.** Formation of zones I, II, III and transition band during normal tear desiccation *in vitro* is an orderly and asynchronous process with only a minor overlapping in the formation of zones II and III. In contrast, production of tear microdesiccates either from normal tear subjected to conditions enhancing evaporation or from tear fluid sampled from patients with Dry Eye may be a quicker process in which major fern-like crystalloids of zones II and III become mostly replaced by homogeneously distributed and synchronously formed groups of small crystalloids. ∆II and ∆III stand for modified zones II and III, respectively.

Based on the dynamics of the drying process we have suggested that accelerated tear water evaporation would be strongly associated with predominant formation of small tear crystalloids. However, such morphological feature of a tear desiccate produced from a given tear sample can be also described as the lack of formation of large crystalloids. This double reading of a single phenomenon with regard to a definite tear sample is necessary due to the possibility of an additional explanation other than the simple higher evaporation rate, as it might be either a deficit or an excess of one or more tear components. Anyhow, regardless the mechanism underlying the organization of tear microdesiccates, a relationship between predominance of small crystalloids in a tear microdesiccate and a higher tear water evaporation rate is highly consistent with an usually observed higher rate of Rolando’s scores III and IV in ferning tests among Dry Eye patients as compared to healthy controls [37, 38]. In the present study, microdesiccates of tear fluid collected from patients with either moderate or severe Dry Eye showed complete absence of major crystalloids. In those cases, the rate of tear water evaporation was about twice the one observed under the same ambient conditions in tear fluid from healthy subjects. Also, time recording of tear desiccation events by direct video imaging and morphological analysis of the resulting tear microdesiccates on samples from both eyes of a patient diagnosed with mild/moderate Dry Eye confirmed both such correspondence between higher rate of desiccation and smaller tear crystalloids as well as the prediction of the mode of formation of the respective microdesiccates. In effect, soon after formation of zone I, many groups of crystalloids started to appear almost synchronously at multiple sites in the still liquid or semiliquid tear material under desiccation. In addition, the growth of those crystalloids was rapidly arrested by contact inhibition. Once again, given the high number of crystalloid origins, the size of each of the resulting crystalloids was markedly smaller. Both this mode of formation and the highly regular small size of the crystalloids are consistent with a high degree of synchrony at the onset of the crystalloid growth. This observation is markedly contrasting with desiccation of normal tear fluid (collected from Non Dry Eye patients) in which formation of major crystalloids of zone II clearly precedes and is only partly overlapped with formation of major crystalloids of zone III (asynchronous processes) (Figure 9). Once more, asynchronous or synchronous formation of major tear crystalloids featuring particular domains of the tear microdesiccates may well be dependent upon the organized (normal) or disorganized (abnormal) distribution of definite tear components that may represent nucleation sites for at least some of the crystalloids. In this regard it is striking the highly regular distribution of the origin sites of zone II crystalloids at specific locations on the transition band in normal tear desiccates [11]. Emerging from those sites, zone II fern-like crystalloids grow centripetally until coming into contact with other tear crystalloids. All these events represent just a minor fraction of an orderly sequence of interactions between tear components. Thus, the whole structure of a tear microdesiccate on a glass surface would be the final expression of a highly organized supramolecular complex whose morphological characteristics would be dependent on *in vitro* intermolecular interactions occurring while tear water is being removed. Some of those characteristics, either normal or abnormal, may be also suggestive of a supramolecular mode of organization of the same tear components structuring the tear film on the wet ocular surface.

## Conclusion

Spontaneous desiccation of a microvolume of tear fluid collected from healthy subjects on a flat glass surface gives rise to tear microdesiccates consisting of well-defined morphological domains. Major fern-like crystalloids are a major feature of some of those domains. Formation of the microdesiccate domains occurs in an orderly sequence of asynchronic events. Under experimental conditions of accelerated desiccation major crystalloids are replaced by smaller crystalloids and asynchrony is lost. The same phenomenon does occur when tear microdesiccates are prepared from tear samples collected from some Dry Eye patients. The observed link between morphological features of tear microdesiccates and relative rate of tear evaporation may provide an additional source of valuable information on tear evaporation from single eyes.

## Methods

### Subjects

All nine healthy subjects (age range 20–59 years old) included in this study fulfilled the following criteria: a) Normal visual parameters, b) Schirmer I test over 10 mm at 5 min [21], c) Fluorescein break-up time over 10 seconds [22], d) OSDI questionnaire scoring below 15 [23], e) Tear ferning test score I or II [5, 6], f) No previous eye surgery and g) No medication during the last three months [4]. In addition, three patients (age range 20–55 years old) diagnosed with Dry Eye according to the DEWS guidelines [8], who had not experienced eye surgery and who were regularly attending the Fundación Oftalmológica Los Andes Ophthalmology Clinic in Santiago, Chile, were invited to be volunteer tear donors. Each one of the diagnostic tests was performed by single trained personnel. The study was conducted between March 2011 and June 2013 in accordance with the tenets of the Helsinki Declaration of 1975 and the guidelines of both the Ethics Committee of the Faculty of Medicine, University of Chile and the Ethics Committee of Fondecyt-Chile (Fondo de Desarrollo Científico y Tecnológico-Chile).

### Tear collection

Tear fluid was collected using polyurethane minisponges, as reported elsewhere [24]. Samples were taken always around 9–11 AM to control for eventual circadian variations. For each eye a single 3-minute tear sample was taken. The amount of sample was determined by gravimetry. Desiccation assays were conducted immediately after tear collection.

### Tear desiccation

Excepting specific experiments (see Results section), one-microliter aliquots of fresh samples of tear fluid were taken with a P2-Gilson micropipette fitted with an ultrafine tip and placed sharply on a point of a microscope slide that was positioned horizontally. Tear aliquots were allowed to dry spontaneously at ambient conditions of temperature (range 15-25°C), relative humidity (range 40-45%) and altitude (520 m above sea level). Micrographs of the dry samples were taken under a dark-field microscope (Zeiss Axiostar Plus, objective lens = 2.5X, ocular lens = 10X) fitted with a Canon Powershot G10 14.7 megapixel digital camera. Fern images were classified as types I through IV according to Rolando’s criteria [5, 6].

### Progression of tear desiccation

Desiccation of one-microliter aliquots of tear fluid was performed under the microscope as described above, except that to this purpose the digital camera was adjusted for capturing high resolution videos. Video-images (30 frames per second) were taken during 1 second at time-intervals of 5 seconds. The endpoint of desiccation was defined as the one in which growth of tear crystalloids was fully halted. Time for full desiccation of each tear sample was scored. In order to prevent any artifactual effect on tear desiccation produced by heat transfer, the lamp of the microscope was turned on only when video-images were being recorded.

### Materials

Absorbing polyurethane mini-sponges (PeleTim®) and microcentrifuge tubes for tear collection were obtained from VOCO, Cuxhaven, Germany and from Axygen Scientific, California, USA, respectively. Sterile Schirmer tear test strips and fluorescein strips were acquired from Alcon Laboratories, Santiago, Chile. Microscope slides were purchased from Isolab Laborgeräte, Wertheim am Main, Germany.

### Consent

Written informed consent was obtained from the patient for the publication of this report and any accompanying images. Confidentiality of patient information was protected.
